# Attitudes and Preparedness of Non-Frontline Physicians in Saudi Arabia Toward the COVID-19 Pandemic

**DOI:** 10.1017/dmp.2021.10

**Published:** 2021-01-08

**Authors:** Haifaa Malaekah, Muna Aljahany, Wajdan Alassaf, Malak Alotaibi, Mashael Alsaffar

**Affiliations:** 1 Department of Surgery, Dr Sulaiman Al Habib Hospital – Altakhassusi, Riyadh, Saudi Arabia; 2 Department of Clinical Sciences, College of Medicine, Princess Nourah Bint Abdulrahman University, Riyadh, Saudi Arabia

**Keywords:** airway management, COVID-19, disaster management, hospital preparedness, personal protective equipment

## Abstract

**Objective:**

This study assesses the attitudes (willingness) and preparedness of non-frontline physicians across different specialties in the Kingdom of Saudi Arabia (KSA) toward the management of hospitalized coronavirus disease (COVID-19) patients.

**Methods:**

This cross-sectional study conducted between April 15, 2020, and May 5, 2020, included 6209 physicians working in KSA. An electronic questionnaire was designed and validated for the assessment of 3 categorical outcome variables, namely, attitudes, confidence, and knowledge levels. Pearson’s chi-square test was used for comparing the distribution of the proportions of these 3 categorical variables.

**Results:**

Most participants (63.2%) were willing and prepared to treat COVID-19 patients. A significantly large proportion of participants specializing in anesthesiology (78.2%) had higher knowledge levels, followed by those from plastic surgery (71.1%), pediatrics (69.7%), and obstetrics and gynecology (69.1%) (*P* < 0.0001). Lower confidence levels were found for airway management skills (38.1%), particularly among dermatologists and radiologists.

**Conclusion:**

Higher knowledge levels about personal protective equipment (PPE) use and confidence in airway management skills were proportionally related to the level of willingness to participate in COVID-19 patient management. There is an urgent need to train doctors from certain specialties on PPE use and airway management to enable their frontline support of severely ill COVID-19 patients.

## Introduction

Severe acute respiratory syndrome coronavirus 2 (SARS-CoV-2), a novel virus belonging to the coronavirus family, was first identified on December 31, 2019, in Wuhan City, Hubei, People’s Republic of China.^1^ In February 2020, the SARS-CoV-2 infection was designated as the coronavirus disease (COVID-19) by the World Health Organization (WHO).^2^ COVID-19 spreads through close and unprotected human–human transmission.^1^ The disease is managed based on the severity of its symptoms. Home management is deemed appropriate for patients with non-severe symptoms (eg, fever, cough, and/or myalgia without dyspnea), who can be adequately isolated in an outpatient setting.^3,4^ Suggested or confirmed COVID-19 patients accompanied by severe symptoms warrant hospitalization. The management of patients with severe disease comprises appropriate infection control and supportive care, including oxygenation support. In such patients, the disease is treated and managed by frontline physicians. In Saudi hospitals, these physicians include emergency physicians, intensivists, pulmonologists, and infectious disease physicians. However, there is an urgent need for assistance from health care professionals across other specialties, owing to the high risk of exposure. For example, during the early stages of the SARS epidemic in Toronto, 60% of the infected health care providers comprised critical care staff.^5^ The WHO recommends multidisciplinary collaboration for disaster mitigation during epidemics. However, several previous studies have found that physicians from different specialties worldwide are unprepared to respond to disasters, owing to a lack of appropriate education, training, or experience.^6,7^ To date, no published study has investigated the knowledge levels, skills, or preparedness of non-frontline physicians in the Kingdom of Saudi Arabia (KSA) in the context of surge management.

Accordingly, this work aimed to study the attitudes (willingness), levels of knowledge on personal protective equipment (PPE) use, and levels of confidence in airway management skills, associated with the COVID-19 pandemic, among non-frontline physicians across the KSA. We believe that this study has the potential to provide basic data for facilitating strategic planning during the pandemic, especially if the current situation worsens, as well as to enable preparedness for future outbreaks.

## Methods

### Study Design and Study Population

In this cross-sectional study conducted between April 15, 2020, and May 5, 2020, non-frontline physicians were enrolled from across the KSA. Physicians who currently reside in the KSA were included, regardless of whether they were currently practicing or retired. Physicians across all clinical specialties were included, except frontline physicians who, by definition, are likelier to directly deal with suggested or confirmed severe COVID-19 cases in Saudi hospitals (ie, emergency physicians, intensivists, pulmonologists, and infectious disease physicians). The sample size was estimated by considering the outcome variables (attitudes, knowledge levels, and confidence levels of non-frontline physicians regarding patient management during the COVID-19 pandemic) as a single composite outcome variable.

### Participant Recruitment

Data were obtained using a self-administered online questionnaire, and participation was voluntary. Participants were recruited through the Saudi Commission for Health Specialties (SCFHS), which is responsible for the supervision and evaluation of training programs, as well as setting of controls and specification of standards for practicing health professionals (licensure). The SCFHS maintains a database of all physicians who live and work in the KSA and facilitates research by the distribution of surveys to health care workers (HCWs). Following the receipt of ethical approval for this study, the Data and Business Department at SCFHS sent out e-mails to all the targeted registered physicians (91 364), followed by a reminder e-mail 2 weeks later. A total of 7696 responses were received; of them, 127 did not agree to participate in the study while 6209 completed the survey.

### Survey Tool

A questionnaire was developed based on information obtained from previous studies,^8,9^ in which surveys were used for assessing the preparedness of physicians toward the SARS and Avian influenza outbreaks, as well as the opinions of 2 national experts. The questionnaire was modified to suit the ongoing COVID-19 pandemic, with the incorporation of additional questions.

The questionnaire was examined further for inadequate expressions or concepts. Additionally, the content of the questionnaire was validated for clarity and adequacy by 2 experts specializing in disaster preparedness and emergency preparedness. Finally, a pilot survey was performed, and the questionnaire was distributed to 27 candidates who represented the study population. The reliability of the questionnaire was assessed using Cronbach’s alpha for the 3 domains, which resulted in attitude, 0.98; confidence, 0.97; and knowledge, 0.96, indicating a high level of internal consistency.

The final version of the questionnaire comprised 5 sections: (1) demographic characteristics, (2) preparedness and willingness to manage patients with COVID-19, (3) confidence in airway management skills, (4) knowledge on PPE use, and (5) willingness to participate in COVID-19 patient management. The section on demographic characteristics included items on sex, age, marital status, and years of experience, among others. In sections 2–4, a linear numeric response format was used. The last section pertained to the physicians’ willingness to participate in patient care, their area of interest, and reasons for their unwillingness to participate.

Twelve items for the evaluation of physicians’ attitudes, 7 for measuring confidence level, and 15 for measuring the knowledge level were employed. In the attitude sections, participants were asked several questions about their involvement in specific training for COVID-19 preparation and their willingness to receive such training. In the confidence of airway management skills section, they were asked about how well they are trained in airway management skills and whether they can participate in airway management teams. In knowledge of PPE use, they were asked about the usefulness of certain measures in the prevention of acquiring COVID-19 (eg, hand hygiene, N95 masks, isolation of patient area, goggles, gowns, and limiting the number of visitors).

All responses were rated on a linear numeric response format. The total attitude, confidence level, and knowledge level scores were in the ranges of 12–60, 7–35, and 15–75, respectively. The total scores were categorized as low, medium, and high based on the percentiles of scores (up to the 25th percentile as low; between 26th to 50th percentile as medium; and > 50th percentile as high), with appropriate cutoffs for attitude levels (≤ 24, 25–36, and > 36, respectively), confidence levels (≤ 14, 15–21, and > 21, respectively), and knowledge levels (≤ 30, 31–45, and > 45, respectively).

### Statistical Analysis

Data were analyzed using SPSS version 26.0 statistical software (IBM Corp, Armonk, NY). A descriptive statistical analysis was performed. Frequencies and percentages were used for the quantification of the categorical study and outcome variables. Pearson’s chi-square test was used to compare the distribution of the proportions of the 3 categorical outcome variables (attitude, confidence levels, and knowledge levels) in relation to the categorical study variables. A *P*-value ≤ 0.05 was considered statistically significant.

### Ethical Considerations

Ethical approval was obtained from the Institutional Review Board of the Princess Nourah Bint Abdulrahman University (IRB log number 20-0136). Only physicians who agreed to participate were directed to questions in the survey tool.

## Results

Table 1 shows the demographic characteristics of the participants. The study sample comprised 6209 participants. The study outcome variables included the physicians’ attitudes (preparedness and willingness) toward the management of COVID-19 patients, confidence in airway management skills, and levels of knowledge on PPE use. Totally, 63.2%, 36.8%, and 68.0% of the participants had high scores regarding their attitudes toward the management of COVID-19 patients, confidence in airway management skills, and knowledge levels on PPE use, respectively.

**Table 1.** Demographic characteristics of the participants (n = 6209)

Characteristics	n (%)
**Age group, years (n = 6206)**	
25–34	1977 (31.9)
35–44	2355 (37.9)
45–54	1202 (19.4)
55–64	580 (9.3)
65–74	92 (1.5)
**Sex**	
Male	4228 (68.1)
Female	1981 (31.9)
**Nationality**	
Saudi	2007 (32.2)
Non-Saudi	4202 (67.7)
**Current relationship status**	
Married, with children	4550 (73.3)
Married, without children	470 (7.6)
Single	1044 (16.8)
Divorced/widowed	145 (2.3)
**People staying with you (n = 6174)**	
Family	5093 (82.5)
Alone	1039 (16.8)
Others	42 (0.7)
**Specialty (n = 6194)**	
Internal medicine (other than that for pulmonary or infectious diseases)	1607 (25.9) 357 (5.8)
Anesthesia	766 (12.4)
Pediatrics	427 (6.9)
Obstetrics and gynecology	724 (11.7)
Family medicine	212 (3.4)
Dermatology	126 (2.0)
Psychiatry	1450 (23.4)
Plastic surgery	525 (8.5)
**Designation (n = 6209)**	
Resident	2698 (43.5)
Fellow	868 (14.0)
Assistant consultant	1034 (16.7)
Associate consultant	296 (4.8)
Consultant	1313 (21.1)
**Current job status (n = 6209)**	
Practicing	6041 (97.3)
Retired	168 (2.7)
**Total years of experience (since starting medical practice) (n = 6111)**	
≤5	1239 (20.3)
6–10	1294 (21.2)
11–15	1284 (21.0)
16–20	1019 (16.7)
>20	1275 (20.9)
**Main hospital care level (n = 5818)**	
Primary	1574 (27.1)
Secondary	1603 (27.6)
Tertiary	1851 (31.8)
More than 1 level	790 (13.6)

COVID-19 = coronavirus disease.

Table 2 shows the association between the participants’ characteristics and their attitudes toward the management of COVID-19 patients. Comparisons between the attitude scores pertaining to the physicians’ preparedness and willingness to manage COVID-19 patients and the study variables showed significant differences for all the study variables, except for the type of hospital in which the participants were working. Participants ages 35–44, 45–54, and 55–64 years and male physicians (65.8%) had significantly higher attitude scores than those in the other age groups and female physicians (*P* < 0.0001). Participants with a current “married, with children” (65.0%) relationship status, those who were staying alone (67.5%), and physicians who were still practicing (63.6%) had significantly higher attitude scores than those with other relationship statuses, physicians staying with family members, and retired physicians, respectively (*P* < 0.0001). In terms of the physicians’ specialties, a significantly large proportion of physicians who specialized in anesthesiology (74.8%) had higher attitude score levels, followed by those specializing in internal medicine (65.3%), pediatrics (65.5%), and plastic surgery (65.2%) (*P* < 0.0001). A significant increasing trend was observed in the attitude scores in relation to the designations of the participants; physicians with designations of assistant consultant, associate consultant, and consultant showed higher attitude scores than those with a designation of a fellow or resident (*P* < 0.0001). A similar increasing trend was observed in the attitude scores in relation to the categories of 5 experience levels (*P* < 0.0001). Physicians working at the secondary care level (67.7%), tertiary care level (69.5%), and at more than 1 care level (69.0%) showed significant higher, positive attitudes than those working at the primary care level (*P* < 0.0001).

**Table 2.** Association between the participants’ characteristics and their attitudes (preparedness and willingness) toward the management of COVID-19 patients

Participants’ Characteristics	Attitude Score Levels			χ^2^-value	*P*-value
	Low n (%)	Moderate n (%)	High n (%)		
**Age groups, years**					
25–34	577 (29.2)	268 (13.6)	1132 (57.2)	62.76	<0.0001
35–44	551 (23.4)	267 (11.3)	1537 (65.3)		
45–54	290 (24.1)	106 (8.8)	806 (67.1)		
55–64	125 (21.6)	62 (10.7)	393 (67.8)		
65–74	21 (22.8)	21 (22.8)	50 (54.1)		
**Sex**					
Male	993 (23.5)	454 (10.7)	2781 (65.8)	39.39	
Female	571 (28.8)	270 (13.6)	1140 (57.5)		<0.0001
**Current relationship status**					
Married, with children	1083 (23.8)	511 (11.2)	2956 (65.0)	28.90	
Married, without children	139 (29.6)	54 (11.5)	277 (58.9)		
Single	299 (28.6)	146 (14.0)	599 (57.4)		<0.0001
Divorced/widowed	43 (29.7)	13 (9.0)	89 (61.4)		
**People staying with you**					
Family	1326 (26)	611 (12.0)	3156 (62.0)	16.61	
Alone	223 (21.5)	104 (10.0)	712 (67.5)		
Others	12 (28.6)	6 (14.3)	24 (57.1)		
**Current job status**					0.002
Still in practice	1500 (24.8)	698 (11.6)	3843 (63.6)	21.22	
Retired	64 (38.1)	26 (15.5)	78 (46.4)		
**Specialty**					
Internal medicine (other than that for pulmonary or infectious disease)	419 (26.1)	139 (8.6)	1049 (65.3)	112.64	<0.0001
Anesthesia	61 (17.1)	29 (8.1)	267 (74.8)		
Pediatrics	179 (23.4)	85 (11.1)	502 (65.5)		<0.0001
Obstetrics and gynecology	97 (22.7)	56 (13.1)	274 (64.2)		
Family medicine	205 (28.3)	90 (12.4)	429 (59.3)		
Dermatology	80 (37.7)	39 (18.4)	93 (43.9)		
Psychiatry	41 (32.5)	24 (19.0)	61 (48.4)		
Plastic surgery	337 (23.2)	168 (11.6)	945 (65.2)		
Others	139 (26.5)	91 (17.3)	295 (56.2)		
**Designation**					
Resident	786 (29.1)	325 (12.0)	1587 (58.8)	63.82	
Fellow	232 (26.7)	99 (11.4)	537 (61.9)		
Assistant consultant	230 (22.2)	114 (11.0)	690 (66.7)		
Associate consultant	69 (23.3)	27 (9.1)	200 (67.8)		
Consultant	247 (18.8)	159 (12.1)	907 (69.1)		<0.0001
**Total years of experience (since starting medical practice)**					
≤5	385 (31.1)	193 (15.6)	661 (53.3)	88.74	
6–10	355 (27.4)	145 (11.2)	794 (61.4)		
11–15	285 (22.2)	146 (11.4)	853 (66.4)		
16–20	230 (22.6)	87 (8.5)	702 (68.9)		
>20	277 (21.7)	142 (11.1)	856 (67.1)		
**Main hospital care level**					<0.0001
Primary	426 (27.0)	220 (14.0)	928 (59.0)	51.91	
Secondary	341 (21.3)	177 (11.0)	1085 (67.7)		
Tertiary	354 (19.1)	211 (11.4)	1286 (69.5)		
More than 1 level	155 (19.6)	90 (11.4)	545 (69.0)		<0.0001

COVID-19 = coronavirus disease.

Table 3 presents a comparison of the 3 levels of confidence in airway management skills based on the characteristics of the participants. Large proportions of participants in the 45–54 and 55–64 years age groups (40.9% and 42.1%, respectively) and male physicians (42.0%) had significantly higher confidence levels than those belonging to other age groups and female physicians (*P* < 0.0001). Participants who were “married, with children” (39.3%), staying alone (44.4%), and still in practice (37.0%), had significantly higher confidence levels than those with other relationship statuses, staying with family members, and retired (*P* < 0.0001 and *P* = 0.009, respectively). A significantly larger proportion of participants with a specialization in anesthesiology (78.4%) had higher confidence levels, followed by those specializing in pediatrics (56.5%) and plastic surgery (40.3%) (*P* < 0.0001). A significant trend was observed between the physicians’ confidence levels and their designations, with larger proportions of assistant consultants (41.9%), consultants (39.1%), and associate consultants (38.2%) showing higher confidence levels than fellows and residents (*P* = 0.001). An increasing trend was observed between the 5 categories of experience level and levels of confidence in airway management skills pertaining to COVID-19 patients, with a larger proportion of participants with greater experience levels showing higher confidence levels (*P* < 0.0001) as well. Participants working in private hospitals (42.4%) had higher confidence levels than those working in other types of hospitals (*P* < 0.0001). Significantly larger proportions of participants working at the secondary care level, tertiary care level, and at more than 1 care level (41.0%, 39.3%, and 41.5%, respectively) had higher confidence levels than participants working at the primary care level (*P* < 0.0001).

**Table 3.** Association between the participants’ characteristics and their levels of confidence in airway management skills in the management of COVID-19 patients

Participants’ Characteristics	Confidence Score Levels			χ^2^-value	*P*-value
	Low n (%)	Moderate n (%)	High n (%)		
**Age groups, years**					
25–34	853 (43.1)	528 (26.8)	596 (30.1)	62.76	<0.0001
35–44	862 (36.6)	570 (24.2)	923 (39.2)		
45–54	421 (35.0)	289 (24.0)	492 (40.9)		
55–64	194 (33.4)	142 (24.5)	244 (42.1)		
65–74	35 (38.0)	27 (29.3)	30 (32.6)		
**Sex**					
Male	1459 (34.5)	993 (23.5)	1776 (42.0)	154.24	
Female	906 (45.7)	564 (28.5)	511 (25.8)		<0.0001
**Current relationship status**					
Married, with children	1638 (36.0)	1122 (24.7)	1790 (39.3)		
Married, without children	195 (41.5)	124 (26.4)	151 (32.1)	52.64	
Single	466 (44.6)	278 (26.6)	300 (28.7)		<0.0001
Divorced/widowed	66 (45.5)	33 (22.8)	46 (31.7)		
**People staying with you**					
Family	2009 (39.4)	1288 (25.3)	1796 (35.3)		
Alone	333 (32.1)	245 (23.6)	461 (44.4)	33.48	
Others	17 (40.5)	12 (28.6)	13 (31.0)		
**Current job status**					<0.0001
Still in practice	2282 (37.8)	1524 (25.2)	2235 (37.0)		
Retired	83 (49.4)	33 (19.6)	52 (31.0)	9.46	
**Specialty**					
Internal medicine (other than that for pulmonary or infectious diseases)	583 (36.3)	425 (26.4)	599 (37.3)		
Anesthesia	73 (20.4)	4 (1.1)	280 (78.4)	769.97	0.009
Pediatrics	224 (29.2)	109 (14.2)	433 (56.5)		
Obstetrics and gynecology	169 (39.6)	160 (37.5)	98 (23.0)		
Family medicine	312 (43.1)	222 (30.7)	190 (26.2)		<0.0001
Dermatology	132 (62.3)	58 (27.4)	22 (10.4)		
Psychiatry	81 (64.3)	32 (25.4)	13 (10.3)		
Plastic surgery	486 (33.5)	379 (26.1)	585 (40.3)		
Others	295 (56.2)	165 (31.4)	65 (12.4)		
**Designation**					
Resident	1087 (40.3)	693 (25.7)	918 (34.0)		
Fellow	337 (38.8)	222 (25.6)	309 (35.6)		
Assistant consultant	356 (34.4)	245 (23.7)	433 (41.9)		
Associate consultant	112 (37.8)	71 (24.0)	113 (38.2)	25.43	
Consultant	473 (36.0)	326 (24.8)	514 (39.1)		
**Total years of experience (since starting medical practice)**					0.001
≤5	567 (45.8)	355 (28.7)	317 (25.6)		
6–10	525 (40.6)	286 (22.1)	483 (37.3)		
11–15	457 (35.6)	327 (25.5)	500 (38.9)		
16–20	361 (35.4)	248 (24.3)	410 (40.2)	105.02	
>20	417 (32.7)	315 (24.7)	543 (42.6)		
**Main hospital care level**					
Primary	633 (40.2)	419 (26.6)	522 (33.2)		<0.0001
Secondary	510 (31.8)	436 (27.2)	657 (41.0)		
Tertiary	660 (35.7)	463 (25.0)	728 (39.3)		
More than 1 level	255 (32.3)	207 (26.2)	328 (41.5)	36.50	<0.0001

COVID-19 = coronavirus disease.

Table 4 shows the association between the participants’ characteristics and their knowledge levels on PPE use in the management of COVID-19 patients. On comparing knowledge levels on PPE use in the management of COVID-19 patients in relation to the study variables, significant differences were observed for all study variables, except for “people staying with you” and “type of hospital in which you are working.” Large proportions of those ages 35–44, 45–54, 55–64, and 65–74 years and male participants (70.5%) had significantly higher PPE knowledge levels than those belonging to other age groups and female participants (*P* < 0.0001). Participants with a current “married, with children” (69.8%) relationship status and who were still practicing (68.7%) had significantly higher knowledge levels than those with other relationship statuses and those who were retired (*P* < 0.0001). In terms of specialization, a significantly larger proportion of participants with a specialization in anesthesiology (78.2%) had high PPE knowledge levels, followed by those specializing in plastic surgery (71.1%), pediatrics (69.7%), and obstetrics and gynecology (69.1%) (*P* < 0.0001). A significant increasing trend was observed between PPE knowledge levels and participants’ clinical designations, with larger proportions of assistant consultants, associate consultants, and consultants showing greater PPE knowledge levels than fellows and residents (*P* < 0.0001). A similar increasing trend was observed between the categories of the 5 experience levels, with higher proportions of participants who had 11–15, 16–20, and > 20 years of total experience (70.7%, 70.9%, and 72.9%, respectively), showing higher knowledge levels than those who had < 5 and 6–10 years of total experience (*P* < 0.0001). Moreover, larger proportions of participants working at the secondary care level, tertiary care level, and at more than 1 care level (72.7%, 73.0%, and 75.1%, respectively) had significantly higher knowledge levels than those working at the primary care level (*P* < 0.0001).

**Table 4.** Association between the participants’ characteristics and their knowledge levels on PPE use in the management of COVID-19 patients

Characteristics	Knowledge Score Levels			χ^2^-value	*P*-value
	Low n (%)	Moderate n (%)	High n (%)		
**Age groups, years**					
25–34	633 (32.0)	89 (4.5)	1255 (63.5)	42.58	<0.0001
35–44	642 (27.3)	59 (2.5)	1654 (70.2)		
45–54	336 (28.0)	28 (2.3)	838 (69.7)		
55–64	141 (24.3)	16 (2.8)	423 (72.9)		
65–74	26 (28.2)	1 (1.1)	65 (70.7)		
**Sex**					
Male	1123 (26.6)	126 (3.0)	2979 (70.5)	30.05	<0.0001
Female	655 (33.1)	67 (3.4)	1259 (63.6)		
**Current relationship status**					
Married, with children	1249 (27.5)	121 (2.7)	3180 (69.8)	29.55	<0.0001
Married, without children	156 (33.2)	15 (3.2)	299 (63.6)		
Single	327 (31.3)	49 (4.7)	668 (64.0)		
Divorced/widowed	46 (31.7)	8 (5.5)	91 (62.8)		
**People staying with you**					
Family	1495 (29.4)	155 (3.0)	3443 (67.6)	8.59	0.072
Alone	265 (25.5)	33 (3.2)	741 (71.3)		
Others	12 (28.6)	3 (7.1)	27 (64.3)		
**Current job status**					
Practicing	1704 (28.2)	187 (3.1)	4150 (68.7)	20.82	<0.0001
Retired	74 (44.0)	6 (3.6)	88 (52.4)		
**Specialty**					
Internal medicine (other than that for pulmonary or infectious diseases)	482 (30.0)	42 (2.6)	1083 (67.4)	63.79	<0.0001
Anesthesia	73 (20.4)	5 (1.4)	279 (78.2)		
Pediatrics	213 (27.8)	19 (2.5)	534 (69.7)		
Obstetrics and gynecology	116 (27.2)	16 (3.7)	295 (69.1)		
Family medicine	231 (31.9)	23 (3.2)	470 (64.9)		
Dermatology	84 (39.6)	5 (2.4)	123 (58.0)		
Psychiatry	46 (36.5)	10 (7.9)	70 (55.6)		
Plastic surgery	366 (25.2)	53 (3.7)	1031 (71.1)		
Others	158 (30.1)	20 (3.8)	347 (66.1)		
**Designation**					
Resident	883 (32.7)	92 (3.4)	1723 (63.9)	67.22	<0.0001
Fellow	263 (30.3)	27 (3.1)	578 (66.6)		
Assistant consultant	273 (26.4)	21 (2.0)	740 (71.6)		
Associate consultant	78 (26.4)	10 (3.4)	208 (70.2)		
Consultant	281 (21.4)	43 (3.3)	989 (75.3)		
**Total years of experience (since starting medical practice)**					
≤ 5	420 (33.9)	55 (4.4)	764 (61.7)	50.95	<0.0001
6–10	394 (30.4)	43 (3.3)	857 (66.3)		
11–15	342 (26.6)	34 (2.6)	908 (70.8)		
16–20	273 (26.7)	24 (2.4)	722 (70.9)		
> 20	318 (24.9)	28 (2.2)	929 (72.9)		
**Main hospital care level**					
Primary	486 (30.9)	46 (2.9)	1042 (66.2)	41.28	<0.0001
Secondary	396 (24.7)	42 (2.6)	1165 (72.7)		
Tertiary	426 (23.0)	74 (4.0)	1351 (73.0)		
More than 1 level	172 (21.7)	25 (3.2)	593 (75.1)		

COVID-19 = coronavirus disease.

### Limitations

This study has certain limitations that must be considered in the interpretation of its results. First, the study sample was not evenly distributed across the specialties. Second, the knowledge levels were assessed based on personal impressions rather than accurate measurements. Further studies assessing the actual knowledge levels and comparing them with HCWs’ willingness to deal with infected patients during epidemics and pandemics should be undertaken. Third, the results have limited generalizability to other populations as this study included HCWs in Saudi Arabia only.

## Discussion

In this study, we aimed to measure the willingness of non-frontline physicians to provide patient care for those with COVID-19, in addition to assessing their knowledge on both airway management skills and proper use of PPE, which are the 2 fundamental components in dealing with COVID-19 cases. This study is the first to investigate the knowledge levels, skills, and preparedness of non-frontline physicians in the KSA, in the context of management of the COVID-19 surge. To the best of our knowledge, this is the first study conducted on non-frontline physicians in Saudi Arabia for this pandemic.

Most of the participants were willing and prepared to participate in COVID-19 patient management (63.2%); this value was higher than that reported in previous studies. One previous study showed that only 53.8% of public health department employees were willing to work during the influenza pandemic.^10^ A similarly lower proportion was reported in another study, in which only 48% of New York City HCWs were willing to work during the SARS outbreak.^11^ Additionally, weather-related disasters and mass casualty events have been shown to be associated with greater levels of willingness to provide care to victims among HCWs than radiological, nuclear, biological, or chemical disasters. The strongest barriers have been identified in association with biological outbreaks.^12^

In the present study, the male sex and living alone were associated with higher willingness levels (65.8% and 67.5%, respectively), consistent with previous findings. Women, in general, showed lower levels of willingness to participate in care provision during disasters (natural, biological, or man-made).^13-15^ Shapira et al. found that female respondents were less willing to work than the male respondents,^12-14^ whereas living alone was associated with a higher willingness, as the risk of infection transmission to family members was low.^12^ Based on these findings, efforts should be made for the provision of training female physicians in dealing with pandemics. Additionally, there is a need for in-depth investigations of the barriers that affect more than 40% of the physician pool, represented by women, for mitigating the related challenges and enhancing the levels of willingness.

The majority of this study’s participants were familiar with the use of PPE (68.3%); this may be correlated with a higher level of willingness to work during the COVID-19 outbreak, as well as trust in work safety, which may have a strong impact on the willingness to participate in disaster management, in general.^15-17^ Therefore, it is recommended that annual training and re-training programs be conducted for HCWs, with a focus on PPE use (pertaining to donning, doffing, and when to use them). Moreover, all health care facilities should have a stockpile of PPE and ensure open communication channels with the infection control department.

The levels of willingness were higher in the 45–64 years age group, as well as among participants with a consultant designation (attending physicians, 69.1%), and those with > 16 years of experience (68.0%). These factors are correlated with higher levels of knowledge and confidence, which may aid HCWs in dealing with a new pandemic. HCWs with greater levels of experience and self-efficacy demonstrated a higher level of willingness to work during influenza pandemics.^18,19^ Interestingly, physicians who were not usually accustomed to dealing with purely medical cases, such as plastic surgeons (65.2%), showed higher willingness levels than family physicians (59.3%).

The participants showed lower levels of confidence in airway management (38.1%), with female physicians and those from certain specialties (dermatology and radiology) showing both lower confidence levels in airway management and lower willingness to manage COVID-19 patients. Since participants with higher scores in the 3 categories displayed more willingness to participate in frontline care of COVID-19 patients, willingness (induced) could be a secondary trait to higher levels of knowledge and confidence. As these scores were highly linked to the physicians’ specialties, those physicians also did not receive urgent training because of their willingness. Therefore, an establishment of a hands-on training program for HCWs in basic and advanced airway management skills, in addition to use of PPE, is recommended from lead agencies.

## Conclusions

Higher levels of knowledge and confidence in airway management are proportionally related to a greater level of willingness to participate in COVID-19 patient management. There is an urgent need for training programs focusing on PPE use and airway management skills for HCWs. Physicians across specialties who are associated with lower levels of contact with critically ill patients, such as those working in the departments of radiology, dermatology, and family medicine, must undergo special training programs to gain knowledge on COVID-19 patient management. Studying the barriers of willingness is a recommended area for future research.

## Supplementary material

For supplementary material accompanying this paper visit http://dx.doi.org/10.1017/dmp.2021.10. click here to view supplementary material
