# Supplementary material for: Attitudes and Preparedness of Non-Frontline Physicians in Saudi Arabia Toward the COVID-19 Pandemic
Source: Disaster Med Public Health Prep. 2021 Jan 8:1–8. doi: 10.1017/dmp.2021.10 (PMC8010289; doi:10.1017/dmp.2021.10)
Supplement: Supplementary file 1 [file dmpsup.zip › S1935789321000100sup003.pdf]

Preparedness and Willingness of Non-Front Line Physicians to Manage Patients with COVID-19 in KSA

Dear Doctor,

Due to the current status of increasing numbers of patients affected by COVID-19, front line physicians dealing with sick patients might need the support of physicians from other health care disciplines. For that, we seek to explore the gaps in knowledge and skills of non-first line specialties in order to better plan and train.

Please answer the following anonymous survey which will take less than 5 min if you are in a specialty other than (Emergency, ICU, ID, pulmonary).

Thank you.

For any inquires please contact us at:
msaljahany@pnu.edu.sa

Do you agree on participation by filling this survey (need 5 minutes)?
○ Yes.
○ No.

SECTION I: Sociodemographic Data

1. What is your age:
   - ○ 25 to 34.
   - ○ 35 to 44.
   - ○ 45 to 54.
   - ○ 55 to 64.
   - ○ 65 to 74.
   - ○ 75 or older.

2. What is your sex:
   - ○ Male.
   - ○ Female.

3. Nationality:
   - ○ Saudi.
   - ○ Non Saudi.

4. Which of the following best describes your current relationship status:
   - ○ Married with children.
   - ○ Married without children.
   - ○ Single.
   - ○ Divorced/ Widow.

5. People staying with you:
   - ○ Family.
   - ○ Alone.
   - ○ Others: ………………………….

6. What is your specialty:
   - ○ Internal medicine (Other than pulmonary or ID).
   - ○ Anesthesia.
   - ○ Pediatrics.
   - ○ Ob/gyne.
   - ○ Family medicine.
   - ○ Dermatology.
   - ○ Psychiatry.
   - ○ Plastic surgery.
   - ○ Orthopedic.
   - ○ ENT.
   - ○ Ophthalmology.
   - ○ General surgery/ subspecialties.
   - ○ Other: …………………....

7. What is your clinical post?
   - ○ Resident.
   - ○ Fellow.
   - ○ Assistant consultant.
   - ○ Associate consultant.
   - ○ Consultant.

8. Years of service at current level:
   …………………………………..…

9. Total years of experience (starting medical practice):
   …………………….....…………

10. What is your current job status?
    - ○ Still in practice.
    - ○ Retired.

11. What is the type of hospital you are currently working in? (You can choose more than one choice)
    - ○ Military.
    - ○ Ministry of Health.
    - ○ University.
    - ○ Private.

12. What is your main hospital care level? (You can choose more than one choice)
    - ○ Primary.
    - ○ Secondary.
    - ○ Tertiary.

13. In which city in KSA are you currently working in?
    …………………………………….

SECTION II: Preparedness and Willingness to Manage Patients with COVID-19

Items	Strongly disagree	Disagree	Neutral	Agree	Strongly agree
I received adequate personal protective equipment use training.					
I was fit tested.					
My institution have clear policies and pathways to deal with COVID-19 cases.					
My institution have clear policies and pathways to deal with COVID-19 surge.					
I have experienced person/department to ask if unsure of use of personal protective equipment.					
I am personally well prepared for COVID-19 outbreak in living conditions level.					
I am able to play a direct role in taking care of patients with COVID-19.					
I am willing to continue to care for patients in the event of COVID-19 outbreak.					
I am willing to take the risk of working with infectious patients.					
I am willing to be involved in crash airway courses to learn and participate in COVID-19 outbreak.					
I am willing to be involved in crash resuscitation courses to learn and participate in COVID-19 outbreak.					
I am willing to be involved in crash management with infectious disease mass causality management courses to learn and participate in COVID-19 outbreak.					

SECTION III: Confidence of Airway Management Skills

Items	Strongly disagree	Disagree	Neutral	Agree	Strongly agree
I am well-trained in basic skills of airway management.					
I can perform intubation independently.					
I can handle different airway instruments.					
I can give medical advice and consultation for patients with compromised airway.					
I can teach different way of airway management to colleagues.					
I am willing to be a part of a team that handles airway management.					
Physicians should be trained in airway management regardless of their specialty.					

SECTION IV: Personal Protective Equipment (PPE)

The following measures are useful in protecting you from contracting COVID-19	Strongly disagree	Disagree	Neutral	Agree	Strongly agree
Area isolation.					
Hand washing.					
Alcohol rubs.					
Prominent notices.					
N95 mask.					
Surgical mask.					
Paper mask.					
Gauze mask.					
Gloves.					
Gowns.					
Goggles.					
Temperature checks.					
Hair covering.					
Shoe covering.					
Limiting visitors.					

SECTION V:

1. Are you willing to participate in management of patients with COVID-19?
   - ○ Yes.
   - ○ No.

2. If your answer is YES, where do you prefer to work in?
   - ○ Emergency department.
   - ○ ICU.
   - ○ Ward.
   - ○ Other: ………………………...

3. If your answer is NO, what is the reason for your wish of no participation?
   - ○ Lack of needed knowledge and skills.
   - ○ Medicolegal.
   - ○ Catching the disease.
   - ○ Infecting loved ones.
   - ○ I don't think I'm needed.
   - ○ Other: ………………………...

4. Do you have any other comments, questions, or concerns?

   …………………………………………………..

   ....

   ....
