# Supplementary material for: Attitudes and Preparedness of Non-Frontline Physicians in Saudi Arabia Toward the COVID-19 Pandemic
Source: Disaster Med Public Health Prep. 2021 Jan 8:1–8. doi: 10.1017/dmp.2021.10 (PMC8010289; doi:10.1017/dmp.2021.10)
Supplement: Supplementary file 1 [file dmpsup.zip › S1935789321000100sup001.doc]

Table.2: Association between study subjects Characteristics and their attitude score levels for preparedness & willingness to manage Covid19 patients (Pearson's residuals)

| Characteristics | Attitude score levels | | |
| --- | --- | --- | --- |
|  | Low | Moderate | High |
| Age groups  25 - 34  35 - 44  45 - 54  55 - 64  65 - 74  Gender  Male  Female  Current relationship status  Married with children  Married without children  Single  Divorced/ Widow  People staying with you  Family  Alone  Others  Current job status  Still in practice  Retired  Specialty  Internal medicine (Other than pulmonary or ID)  Anesthesia  Pediatrics  Ob/gyne  Family medicine  Dermatology  Psychiatry  Plastic surgery  Others  Type of clinical post  Resident  Fellow  Assistant consultant  Associate consultant  Consultant  Total years of experience (since medical practice)  <=5  6-10  11-15  16-20  >20  Main hospital care level  Primary  Secondary  Tertiary  More than one level | 4.9  -2.6  -1.0  -2.1  -0.5  -4.5  4.5  -4.2  2.3  2.8  1.3  3.0  -3.1  -0.5  3.9  -3.9  1.0  -3.6  -1.2  -1.2  2.1  4.3  1.9  -1.9  0.7  6.3  1.1  -2.4  -0.8  -6.0  5.5  2.2  -2.7  -2.0  -3.1  5.8  -0.7  -3.5  -1.7 | 3.2  -0.6  -3.4  -0.8  3.4  -3.3  3.3  -1.7  -0.1  2.6  -1.0  1.7  -1`.8  0.5  1.6  -1.6  -4.3  -2.1  -0.5  1.0  0.7  3.1  2.6  -0.1  4.3  -0.8  -0.3  -0.7  -1.4  0.6  4.8  -0.6  -0.4  -3.4  -0.7  2.8  -1.4  -1.0  -0.6 | -6.6  2.7  3.1  2.4  -1.8  6.3  -6.3  4.9  -2.0  -4.2  -0.4  -3.8  4.0  -0.8  -4.6  4.6  2.0  4.7  1.4  0.4  -2.3  -5.9  -3.5  1.8  -3.5  -6.2  -0.8  2.6  1.6  5.0  -8.1  -1,6  2.7  4.1  3.2  -7.0  1.6  3.7  1.9 |

Table.3: Association between study subjects Characteristics and their confidence score levels towards airway management skills in the management Covid19 patients (Pearson's residuals)

| Characteristics | Confidence score levels | | |
| --- | --- | --- | --- |
|  | Low | Moderate | High |
| Age groups  25 - 34  35 - 44  45 - 54  55 - 64  65 - 74  Gender  Male  Female  Current relationship status  Married with children  Married without children  Single  Divorced/ Widow  People staying with you  Family  Alone  Others  Current job status  Still in practice  Retired  Specialty  Internal medicine (Other than pulmonary or ID)  Anesthesia  Pediatrics  Ob/gyne  Family medicine  Dermatology  Psychiatry  Plastic surgery  Others  Type of clinical post  Resident  Fellow  Assistant consultant  Associate consultant  Consultant  Total years of experience (since medical practice)  <=5  6-10  11-15  16-20  >20  Main hospital care level  Primary  Secondary  Tertiary  More than one level | 5.6  -1.9  -2.5  -2.4  0.0  -8.5  8.5  -5.6  1.6  4.8  1.9  4.3  -4.5  0.3  3.1  -3.1  -1.7  -7.0  -5.3  -0.7  3.0  7.4  6.1  -4.0  9.0  3.1  0.5  -2.7  -0.1  -1.7  6.2  2.1  -2.1  -1.9  -4.4  4.7  -3.5  0.3  -2.0 | 2.0  -1.2  -0.9  -0.3  1.0  -4.2  4.2  -1.3  0.7  1.3  -0.7  1.0  -1.2  0.5  -1.6  1.6  1.5  -10.8  -7.4  6.1  3.7  0.8  0.1  1.1  3.5  1.0  0.4  -1.1  -0.4  -0.2  3.3  -2.8  0.4  -0.6  -0.3  0.4  1.1  -1.4  0.0 | -7.5  3.0  3.3  2.8  -0.8  12.3  -12.3  6.8  -2.2  -5.9  -1.3  -5.3  5.6  -0.8  -1.6  1.6  0.4  16.8  12.0  -6.2  -6.3  -8.1  -6.2  3.1  -12.2  -4.0  -0.8  3.7  0.5  2.0  -9.2  0.4  1.7  2.4  4.8  -5.0  2.5  1.0  1.9 |

Table.4: Association between study subjects Characteristics and their knowledge score levels towards the utility of personal preparedness equipments in the management Covid19 patients (Pearson's residuals)

| Characteristics | Knowledge score levels | | |
| --- | --- | --- | --- |
|  | Low | Moderate | High |
| Age groups  25 - 34  35 - 44  45 - 54  55 - 64  65 - 74  Gender  Male  Female  Current relationship status  Married with children  Married without children  Single  Divorced/ Widow  People staying with you  Family  Alone  Others  Current job status  Still in practice  Retired  Specialty  Internal medicine (Other than pulmonary or ID)  Anesthesia  Pediatrics  Ob/gyne  Family medicine  Dermatology  Psychiatry  Plastic surgery  Others  Type of clinical post  Resident  Fellow  Assistant consultant  Associate consultant  Consultant  Total years of experience (starting medical practice)  <=5  6-10  11-15  16-20  >20  Main hospital care level  Primary  Secondary  Tertiary  More than one level | 4.0  -1.9  -0.6  -2.4  -0.1  -5.3  5.3  -3.4  2.3  2.1  0.8  2.5  -2.5  0  4.5  -4.5  1.5  -3.5  -0.5  -0.7  2.1  3.6  2.0  -3.2  0.8  6.3  1.2  -1.7  -0.9  -6.5  4.6  1.7  -1.7  -1.4  -3.2  5.8  -0.8  -2.9  -2.5 | 4.3  -2.1  -1.7  -0.5  -1.1  -0.9  0.9  -3.4  0.1  3.2  1.7  -0.5  0.2  1.5  0.4  -0.4  -1.3  -1.9  -1.1  0.8  0.1  -0.6  3.1  1.4  1.0  1.2  0.0  -2.2  0.3  0.4  3.3  0.7  -0.9  -1.3  -1.9  -0.8  -1.6  2.3  -0.1 | -5.5  2.6  1.2  2.5  0.5  5.4  -5.4  4.6  -2.2  -3.3  -1.4  -2.2  2.4  -0.5  -4.5  4.5  -0.9  4.1  0.9  0.4  -2.1  -3.3  -3.1  2.6  -1.1  -6.5  -1.1  2.5  0.8  6.2  -5.7  -1.9  2.0  1.8  3.9  -5.3  1.4  1.9  2.5 |
